# Epilepsy in low- to middle-income countries

**DOI:** 10.1097/WCO.0000000000001350

**Published:** 2025-02-10

**Authors:** Arjune Sen, Charles R. Newton, Gift Ngwende

**Affiliations:** a Oxford Epilepsy Research Group, Nuffield Department of Clinical Neurosciences, John Radcliffe Hospital; b Centre for Global Epilepsy, Wolfson College; c Department of Psychiatry, Warneford Hospital, University of Oxford, Oxford, UK; d Neurosciences Unit, Kenya Medical Research Programme-Wellcome Trust Collaborative Programme, Kilifi, Kenya; e Department of Medicine, University of Zimbabwe, Harare, Zimbabwe

**Keywords:** diagnostic gaps, stigma, technological solutions, treatment gap, WHO Intersectoral Global Action Plan

## Abstract

**Purpose of review:**

Epilepsy disproportionately affects those in low- and middle-income countries (LMICs) where diagnostic and treatment gaps persist.

We highlight key recent developments and showcase practical opportunities to improve epilepsy care in resource limited settings.

**Recent findings:**

In LMICs, cultural, socioeconomic and infrastructural factors drive the epilepsy treatment gap. Robust implementation of the WHO Intersectoral Global Action Plan (WHO IGAP) and Mental Health Gap Action Program (mhGAP), for example, will reduce the epilepsy education gap. Engaging traditional healers and other key community leaders should lessen stigma. The Epilepsy Diagnostic Companion, a culture specific tool that helps identify convulsive seizures, can expedite epilepsy diagnosis at primary care level. Novel, robust 3-D printable EEG headsets prototypes that can be deployed in remote rural communities have been piloted with encouraging results. Levetiracetam has been added to the WHO Essential Medicines List (EML), paving way to safer, less teratogenic antiseizure medications (ASMs). Epilepsy surgery programs in carefully selected patients potentially offer cheap, effective and potentially curative treatments, including in LMICs.

**Summary:**

Apps, EEG prototypes, better access to ASMs and implementation of WHO iGAP offer current, tangible opportunities to improve epilepsy care in LMICs. Bidirectional learning must be facilitated to also help hard to reach communities in high-income settings.

## INTRODUCTION

Epilepsy is a condition of inequity. The disorder is disproportionately over-represented in low- to middle-income countries (LMICs), particularly sub-Saharan Africa and some regions of South America [1]. For example, around 10 million people with epilepsy live in Africa alone.

The prevalence of epilepsy will also increase substantially over the next few decades. Populations are growing and those with early onset epilepsy are thankfully living into later life. Although epilepsy affects people across their life course, the highest incidence is in younger children and after the age of 55 years [2]. The rate of ageing is markedly higher in LMICs [3]. Therefore, there is an impending ‘tsunami of need’ to deliver more epilepsy care with this impact being felt most acutely in countries where there is least infrastructure to manage that need.

Diagnostic and treatment gaps are further compounded by the deep stigmatisation that surrounds epilepsy. Enactment of that stigma differs across countries. Why, though, a condition that was described in Babylonian texts [4] is still so poorly misunderstood is a source of endless bafflement.

Taking all of this into account, the World Health Organization (WHO) chose epilepsy as its lead tracer condition in the recent Intersectoral Global Action Plan (IGAP) [5^▪▪^]. Epilepsy is highlighted in recent versions of WHO mhGAP [6^▪▪^] and the antiseizure medication (ASM) levetiracetam has been added to the WHO Essential Medicines List [7^▪▪^]. Researchers from across the world have also begun to galvanise around large studies exploring epilepsy in LMICs.

In this article, we highlight key recent developments relating to epilepsy in resource limited settings and showcase clear opportunities that can now be leveraged to improve the care of the many people with epilepsy who live in LMICs. 

**Box 1:**
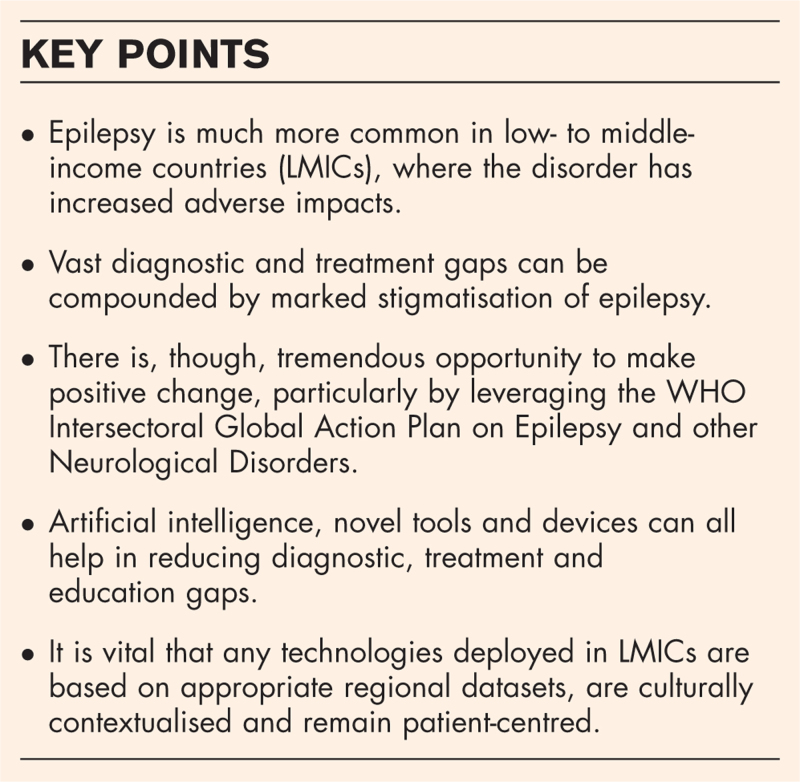
no caption available

## EPIDEMIOLOGY OF EPILEPSY GLOBALLY

**FIGURE 1:**
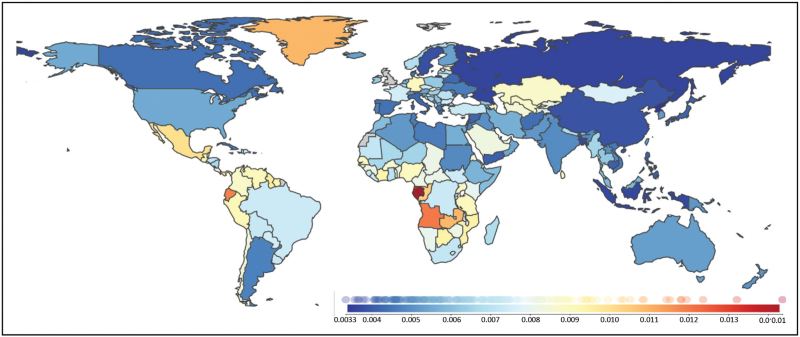
Prevalence of epilepsy in males in 2021. Overall more of the world's population has epilepsy, although this is distributed variably. Notably, the IHME continues to classify epilepsy as Idiopathic (no cause found) or secondary, despite changes in classification by the International League Against Epilepsy (ILAE). This allows comparisons to be made with the estimates in 1990 and the intervening years *Institute for Health Metrics and Evaluation (IHME). GBD Results. Seattle, WA: IHME, University of Washington, 2024. Available from*https://vizhub.healthdata.org/gbd-results/*(Accessed 8*^*th*^* November 2024]). Source: Institute for Health Metrics and Evaluation. Used with permission. All rights reserved*.

The most recent estimates of the global burden of epilepsy come from the Institute of Health Metrics and Evaluation (IHME) at the University of Washington. In 2021, the IHME estimated that there were 51.7 million people (95% Uncertainty Interval 44.9–58.9), 0.7% of the world's population, with active epilepsy [8] (Fig. 1).

There has been an increase in epilepsy owing to secondary causes and this has focused attention on primary prevention. In LMICs, perinatal problems, infections of the central nervous system and traumatic brain injury are the main risk factors for the development of epilepsy [1]. Better treatment for HIV has also been associated with the later emergence of HIV-Associated Neurocognitive Disorder (HAND) a component of which is epilepsy [9]. Recently there are reports demonstrating that control of onchocerciasis is associated with 70% reduction in the incidence of epilepsy in South Sudan [10] and southern Tanzania [11]. The impact of better perinatal care and reduction of traumatic brain injury on epilepsy is unknown, but it has to be anticipated that improvements in these areas will be beneficial.

Work should also address vascular risk factors. Changes in lifestyle and ageing demographics in LMICs lead to increased prevalence of stroke and dementia. The most common cause of later onset epilepsy (over the age of 55 years) is stroke [12]. Tackling vascular risk factors in mid-life may therefore mitigate the risk of both stroke and thereby poststroke epilepsy. Whether addressing vascular risk factors can reduce the risk of epilepsy in those who have not had a stroke warrants urgent investigation.

## PROGNOSIS OF UNDIAGNOSED, UNTREATED AND POORLY TREATED EPILEPSY

Seizures in LMICs impose a significant social and health burden. The vast epilepsy management gap is associated with excess early mortality, with standardised mortality rates that are six times higher in LMICs compared to HICs [13–15]. The annual mortality rate in those who have epilepsy is about three times compared to those without epilepsy [16]. This excess in premature mortality is driven by multiple factors that include direct causes such as Sudden Unexpected Death in Epilepsy (SUDEP) and complications of status epilepticus – which is often managed in ill-equipped health facilities. Indirect excess premature mortality in LMICs includes deliberate self-harm and unintentional injuries such as burns, traumatic brain injuries and drowning [17]. The social consequences of untreated or sub-optimally treated epilepsy include poor quality of life, stigma and employment discrimination in economies that already have a high unemployment rate. Educational opportunities can be limited owing to stigma and the prospects for long lasting relationships are significantly reduced [18,19]. The solutions to reducing the poor prognosis in epilepsy in LMIC therefore lie with holistic programmes, interventions and research that addresses gaps in epilepsy care.

## DIAGNOSTIC AND TREATMENT GAPS IN EPILEPSY

The WHO reported that in 2017 there was a median of 0.1 neurologists per 100 000 population in LMICs compared with 7.1 per 100 000 population in high-income countries [20]. This 70-fold discrepancy poses incredible strain on the healthcare systems of LMICs.

As well as the lack of clinicians skilled in the diagnosis of epilepsy, LMICs have very limited access to diagnostic tools such as brain imaging and electroencephalograms (EEGs). The ‘diagnostic gap’ is matched by a vast ‘treatment gap’. The treatment gap, in turn, exacerbates the risk from uncontrolled seizures – often in environments where having a seizure is intrinsically more dangerous. For example, if clothes must be washed in running water or food cooked on open fires, there is much more inherent risk from any episode of loss of consciousness.

## HOW DO WE SOLVE THIS ‘TSUNAMI OF NEED’?

Whilst care needs relating to epilepsy in LMICs can seem overwhelming, there are many current opportunities.

## EDUCATION GAPS

WHO IGAP [5^▪▪^] has the possibility to transform the lived experience for those with epilepsy, particularly as there is a specific goal directed to improving the public healthcare delivered for those with seizures (WHO IGAP goal 5). Similarly, there have been revisions to the WHO mhGAP [6^▪▪^]. To be meaningful, such updated guidance requires iterative training and dissemination, especially to primary healthcare workers.

## DIAGNOSTIC GAPS

### Apps

**FIGURE 2:**
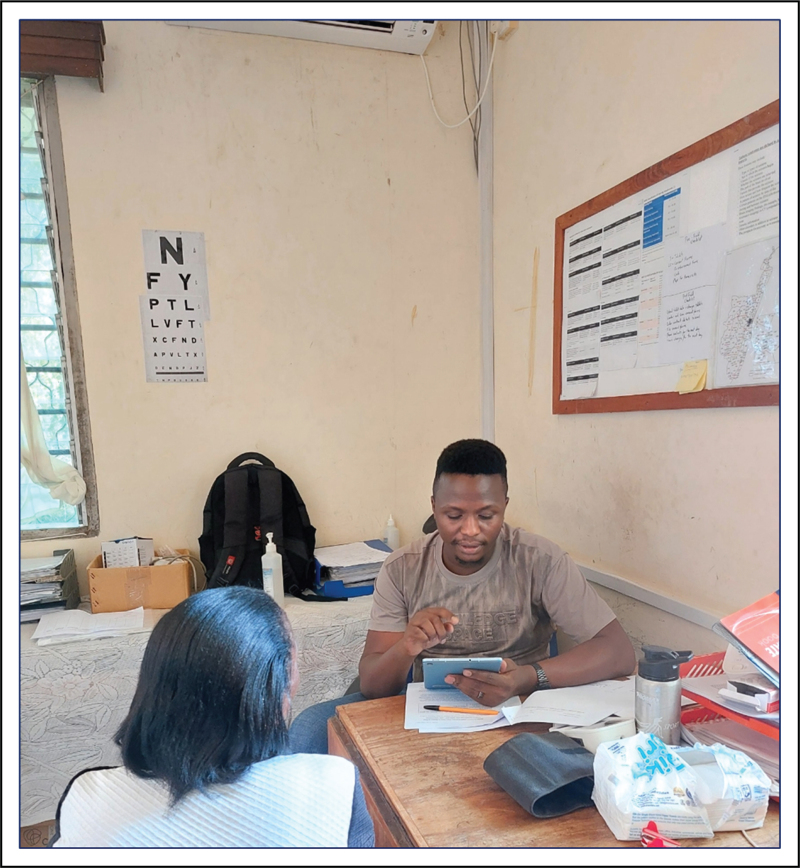
Apps and technologies offer a potential solution to the vast needs in epilepsy care across LMICs. Clinicians, primary healthcare workers and pharmacists can all leverage from the rapid evolution of healthcare technologies. It will remain essential that any deployed technology is appropriately contextualised and subjected to regular audits of safety and efficacy (photo: Mercy Atieno, with permission).

There has been considerable work on apps to help primary care workers streamline pathways. For example, the Epilepsy Pathway Innovation in Africa (EPInA) study group [21] and the Oxford Martin Programme on Global Epilepsy [22] have developed and validated the Epilepsy Diagnostic Companion (EDC), a culture specific tool for rapid detection of patients with convulsive seizures [23^▪^] (Fig. 2). Retesting the EDC in sites that contributed to the datasets show that there can be substantial variability in sensitivity/specificity even within a region [24].

We cannot emphasise strongly enough how important it is that apps are based on large locally acquired datasets, are culturally contextualised and are validated in the settings in which they will operate. Deploying apps that have been developed elsewhere into new regions cannot be recommended unless substantial validation is proven. In countries where technology access can be limited, poor technology can do more harm than good.

### Electroencephalography

Concentrated efforts are also being made to develop more robust electroencephalogram (EEG) headsets that can be deployed to remote rural communities where the largest epilepsy burden manifests. EEGs are a key diagnostic tool that ensures accurate characterisation of electroclinical epilepsy syndromes. Currently, EEG machines are few, delicate and unsuitable for remote use. Most EEGs are only available in urban areas leading with most patients travelling hundreds of kilometres in areas with poor road networks for routine inter-ictal recordings. The cost of a routine EEG remains prohibitive in regions where resources are already scarce with figures ranging from US$50–US$100 [25^▪^].

Epilepsy researchers have developed EEG headsets designed for ease of use by less trained healthcare workers and suitable for use in remote areas [26]. One such EEG headset prototype is the AGENDA EEG headset which has been developed with the active participation of epilepsy researchers and people living with epilepsy in Brazil, Kenya, India, South Africa and Zimbabwe [22]. Other than the robustness of the prototype and relative simplicity of use, the AGENDA EEG uses a straightforward classifier algorithm that allows diagnosis of epileptiform discharges looking to only classify whether discharges are generalised or restricted to one brain region. Several components of this novel EEG, including the electrodes, are 3-D printable, making rapid local production feasible. Other colleagues are developing culturally suitable EEG electrodes that are suitable for use in patients with coarse hair, which is the commonest hair type on the African continent [27] and there is much effort to create/develop algorithms to read the EEGs from such headsets also. Collaborative and transparent shared working will be essential in this space to avoid duplication and redundancy.

### Magnetic resonance imaging

**FIGURE 3:**
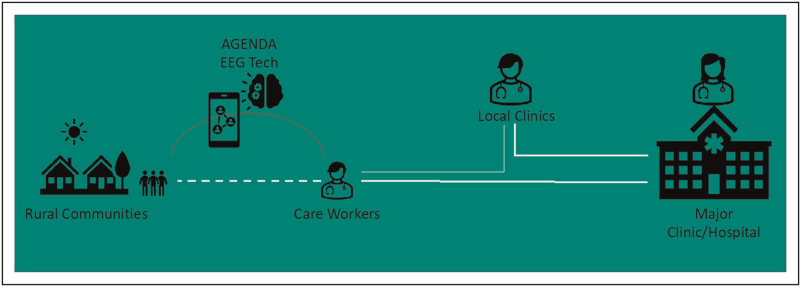
Potential intervention points to streamline access through LMIC healthcare pathways. Empowering primary healthcare workers with culturally contextualised technologies will enable better and more accurate diagnosis of epilepsy closer to communities. Appropriate onward referral can then be more assured – enabling the right person to be seen by the right clinician. This will reduce pressure through the entire care pathway as those with greatest need can be prioritised for brain imaging and hospital care (Infographic courtesy of Oxford Martin Programme on Global Epilepsy).

Although not portable, which potentially reduces scalable applicability for MRI, low field MRI has improved dramatically over recent years and can be a very helpful diagnostic tool in LMICs [28]. A pathway that we envisage would enable widescale EEG recording for people who have experienced a potential seizure, this also being utilised as a stratification tool to determine which individuals would most benefit from referral to a hospital and to be considered for brain imaging (Fig. 3).

## TREATMENT GAPS

Once a diagnosis of epilepsy is made, especially in LMICs, there must be treatment options available. Otherwise, there is a risk that people feel ‘labelled’ with a potentially stigmatising condition but have no solutions.

### Medications

Often, only 10–15% of people living with epilepsy in LMICs receive a regular supply of ASMs. Older ASMs, which are usually the only ones available in LMICs (Table 1), have multiple short- and long-term adverse effects including longer term risks to bone health and cholesterol metabolism. Several of the older ASMs interact with commonly used drugs such as antiretrovirals. Also, many of these ASMs, most notably sodium valproate, associate with teratogenic risk. To address this, levetiracetam was added to the WHO Essential Medicines List (EML) [7^▪▪^]. Levetiracetam has few drug-drug interactions and the lowest teratogenic risk of all ASMs. It is now crucial that levetiracetam is added to national EMLs and that the medication, as well as other treatments on the WHO EML, are made available, accessible and affordable for local populations [29^▪^].

**Table 1:** Availability of ASM in Zimbabwe at central and provincial hospitals as of 29 May 2024

	PH	CBZ	PHT	LTG	VAL	LEV	DZP	LZP
Parienyatwa	√	x	√	x	x	√	x	x
SMCH	√	x	√	x	x	x	√	x
UBH	√	x	√	x	x	x	x	x
Chitungwiza	X	√	√	x	x	x	√	x
Gweru	X	√	x	√	√	x	x	x
Mpilo	√	x	√	x	√	x	x	x
Mutare	X	x	x	√	x	x	√	x
Masvingo	X	x	x	x	x	√	x	x

The Zimbabwean League Against Epilepsy (ZLAE) surveyed all major hospitals in Zimbabwe to assess their availability of key ASMs in May 2024. There was wide variability in the medications available with phenytoin and phenobarbitone being most widely prescribable. CBZ, carbamazepine; DZP, diazepam; LEV, levetiracetam; LTG, lamotrigine; LZP, lorazepam; PH, phenobarbitone; PHT, phenytoin; SMCH, Sally Mugabe Central Hospital; UBH, United Bulawayo Hospital; VAL, sodium valproate.

Stockouts, other disruptions to ASM pipelines and high cost can all contribute to lack of medication adherence. Imparting knowledge about the importance of concordance is vital as is patient empowerment. Systems, for example, short messaging service (SMS), can offer reminders to people with epilepsy to take ASMs and may enable quantitative documentation of concordance prior to clinic visits.

### Epilepsy surgery

Although on first glance paradoxical, it could be argued that epilepsy surgery – which is of proven clinical efficacy – is more needed in LMICs than in high income settings as it offers the possibility of seizure remission with, perhaps, less need for ASMs [30]. Data on epilepsy surgery in LMICs are limited, although reported outcomes are favourable [31–34]. This could, of course, represent publication bias.

Nonetheless, with improvements in MRI and surgical skills, epilepsy surgery might offer a very promising avenue to help ‘cure’ certain epilepsies in LMICs. Whilst in high-income settings, unilateral hippocampal sclerosis is now relatively uncommon in surgical programmes, this need is still very evident in sub-Saharan Africa. Costs for epilepsy surgery can be far less, in absolute terms, than in higher income countries, but those costs would still represent a substantial part of LMIC health budgets where monies allocated to health can be very limited [35]. Ensuring all aspects of the epilepsy surgery programme are recapitulated in LMICs, for example neuropsychometry and neuropsychiatric testing, will also be important to select optimal resection candidates and to mitigate postoperative risk.

## REDUCING STIGMA

Underpinning diagnostic, treatment and education gaps is stigma. Much of the stigma around epilepsy in the LMICs is driven by deep seated traditional and cultural beliefs. In many LMIC societies, the first port of call for both consultation and treatment of epilepsy are traditional healers or religious leaders whose explanations are culturally acceptable [36]. A recent study found out that the use of traditional methods to treat epilepsy leads to a diagnostic and treatment delay of up to 3 years [37]. Reaching out to these frontline key community leaders greatly increases societal understanding of epilepsy and leads to increased referrals to epilepsy treatment centres.

One novel way to increase epilepsy awareness, reduce real and perceived stigma and ultimately achieve WHO IGAP objectives is the use of dedicated epilepsy awareness days/weeks [38^▪^]. Progress has also been achieved through the use of socially and culturally appropriate awareness campaigns and specific destigmatization tool kits designed by the International Bureau for Epilepsy (IBE) and International League Against Epilepsy (ILAE) in conjunction with those living with epilepsy in Africa [39]. A recent systemic review identified four broad interventions aimed at reducing stigma, but three quarters of the studies included came from HICs making it difficult to generalise those conclusions to LMIC settings [40]. It, therefore, still today, remains essential to capture the lived experiences of people with epilepsy in LMICs and better understand in-country experience of stigma.

## BIDIRECTIONAL LEARNING

Whilst we have focussed on epilepsy in LMICs, it is important to fully recognise that care is not evenly distributed through high income settings. Great inequity exists here also. For example, access to tertiary epilepsy centres can vary substantially across the United Kingdom [41]. Availability of diagnostic tests can also be similar to LMICs for certain populations in higher income settings. For example, in the Kivalliq region of the Canadian Arctic, there can be high rates of status epilepticus yet the average time from seizure onset to EEG was 3.2 days as all cases required airlifting to a hospital 1200–1900 km away [42].

WHO IGAP applies to all member states, not just LMICs. There is clear opportunity, and need, to share learning globally to drive improvements in epilepsy care for all hard to reach communities.

## CONCLUSIONS AND FUTURE DEVELOPMENTS

Whilst the evident need to improve things for people with epilepsy is immense, there is, as we have sought to highlight, enormous current opportunity.

As well as leveraging best practice from WHO IGAP/mhGAP, given the limited clinical resources technological solutions will be essential. Apps to help better diagnose convulsive epilepsy are already deployed and, for example, development of low field MRI and portable, lightweight and durable EEG headsets all offer great potential. It is, though, vital that technologies are informed by relevant data and are culturally contextualised with true community engagement. Whilst this will help sustainability of new devices and apps, iterative working with local stakeholders will remain essential to ensure that technologies become fully embedded into in-country health infrastructure.

## Acknowledgements

*This research was commissioned by the National Institute for Health Research (grant number NIHR200134) using Official Development Assistance (ODA) funding. The views expressed in this publication are those of the author(s) and not necessarily those of the NHS, the National Institute for Health Research or the department of Health and Social Care.*

### Financial support and sponsorship

*None.*

### Conflicts of interest

*There are no conflicts of interest.*

## REFERENCES AND RECOMMENDED READING

Papers of particular interest, published within the annual period of review, have been highlighted as:

▪ of special interest
▪▪ of outstanding interest
